# Microbial Community Composition of Polyhydroxyalkanoate-Accumulating Organisms in Full-Scale Wastewater Treatment Plants Operated in Fully Aerobic Mode

**DOI:** 10.1264/jsme2.ME12141

**Published:** 2012-12-19

**Authors:** Mamoru Oshiki, Motoharu Onuki, Hiroyasu Satoh, Takashi Mino

**Affiliations:** 1 Division of Environmental Engineering, Faculty of Engineering, Hokkaido University, North-13, West-8, Sapporo, Hokkaido 060–8628, Japan; 2 Institute of Environmental Studies, School of Frontier Sciences, The University of Tokyo, 5–1–5 Kashiwanoha, Kashiwa, Chiba 277–0882, Japan

**Keywords:** activated sludge, fluorescence *in situ* hybridization, microbial community composition, polyhydroxyalkanoates-accumulating organisms, wastewater treatment plants

## Abstract

The removal of biodegradable organic matter is one of the most important objectives in biological wastewater treatments. Polyhydroxyalkanoate (PHA)-accumulating organisms (PHAAOs) significantly contribute to the removal of biodegradable organic matter; however, their microbial community composition is mostly unknown. In the present study, the microbial community composition of PHAAOs was investigated at 8 full-scale wastewater treatment plants (WWTPs), operated in fully aerobic mode, by fluorescence *in situ* hybridization (FISH) analysis and post-FISH Nile blue A (NBA) staining techniques. Our results demonstrated that 1) PHAAOs were in the range of 11–18% in the total number of cells, and 2) the microbial community composition of PHAAOs was similar at the bacterial domain/phylum/class/order level among the 8 full-scale WWTPs, and dominant PHAAOs were members of the class *Alphaproteobacteria* and *Betaproteobacteria*. The microbial community composition of *α*- and *β*-proteobacterial PHAAOs was examined by 16S rRNA gene clone library analysis and further by applying a set of newly designed oligonucleotide probes targeting 16S rRNA gene sequences of *α*- or *β*-proteobacterial PHAAOs. The results demonstrated that the microbial community composition of PHAAOs differed in the class *Alphaproteobacteria* and *Betaproteobacteria*, which possibly resulted in a different PHA accumulation capacity among the WWTPs (8.5–38.2 mg-C g-VSS^−1^ h^−1^). The present study extended the knowledge of the microbial diversity of PHAAOs in full-scale WWTPs operated in fully aerobic mode.

Polyhydroxyalkanoates (PHA) are polyesters found in microbial cells as carbon and energy storage materials (5, 29). To date, PHA accumulation by microorganisms has been identified in various natural and engineered ecosystems such as soil (28, 53), compost (53), river biofilm (17), marsh microbial mats (53), algal mat (10), estuarine sediment (16, 21, 45), freshwater (36, 60) and activated sludge processes (45, 53, 63).

A variety of PHA-accumulating organisms (PHAAOs) have been identified in activated sludge processes so far. It is well known that the members of polyphosphate-accumulating organisms (PAOs) and glycogen-accumulating organisms (GAOs) take up organic matter and concomitantly synthesize PHA under anaerobic conditions (42). As a member of PAOs or GAOs, PHA accumulation has been identified in *Candidatus* “Accumulibacter phosphatis” (18, 22, 46), *Candidatus* “Competibacter phosphatis” (44) and *Defluvicoccus*-related microorganisms (32, 64). Other than PAOs or GAOs, filamentous bacteria such as *Sphaerotilus natans* have also been identified as PHAAOs in activated sludge; however, the outline of microbial community composition of PHAAOs is still unknown in activated sludge processes since previous studies have just focused on the individual members of PHAAOs.

The present study was conducted to investigate the microbial community composition of PHAAOs in activated sludge. For this purpose, activated sludge samples were collected from 8 full-scale wastewater treatment plants (WWTPs) operated in fully aerobic mode. The activated sludge samples were incubated with the addition of acetate to allow PHAAOs to accumulate PHA, and then subjected to the following microbial community structure analysis. First, the abundance of PHAAOs was enumerated using microscopy subsequent to dual staining with 4′,6-diamidino-2-phenylindole (DAPI) and Nile blue A (NBA), a specific fluorescence dye for PHA granules (50). Next, microbial community compositions of PHAAOs were investigated by fluorescence *in situ* hybridization (FISH) analysis with bacterial domain/phylum/class/order-specific oligonucleotide probes. The accumulation of PHA in the cells hybridized with an oligonucleotide probe was subsequently identified by post-FISH NBA staining technique (27, 56). Moreover, 16S rRNA gene cloning libraries were generated from the activated sludge samples, 16S rRNA gene sequences determined, and 4 and 12 oligonucleotide probes designed, targeting *α*- and *β*-proteobacterial PHAAOs, respectively. The designed oligonucleotide probes were applied to the activated sludge samples to further examine the microbial community composition of *α*- or *β*-proteobacterial PHAAOs.

## Materials and Methods

### Activated sludge

Activated sludge samples were collected from 8 municipal WWTPs in Japan. These WWTPs were operated in fully aerobic mode where the reaction tank was mixed with air aeration. The concentration of dissolved oxygen in the reaction tanks was maintained at the range of 1 to 3 mg O_2_ L^−1^ to maintain the aerobic condition. The operational conditions of the 8 WWTPs are summarized in Table S1. One liter of biomass suspension was collected from the end of the aeration tank and taken to the laboratory with cooling on ice. The activated sludge samples obtained from A WWTP to H WWTP are hereafter referred as AS-A to AS-H, respectively.

### Batch experiments

The activated sludge samples were washed twice with inorganic media (CaCl_2_·2H_2_O 44 mg L^−1^, MgCl_2_·6H_2_O 453.5 mg L^−1^, KCl 210 mg L^−1^, NH_4_Cl 88 mg L^−1^, (NH_4_)_2_SO_4_ 108 mg L^−1^, K_2_HPO_4_ 90 mg L^−1^, and KH_2_PO_4_ 70 mg L^−1^), and the concentration of mixed liquor volatile suspended solids (MLVSS) was set at 500 mg L^−1^. The biomass suspension was aerobically incubated at 22°C for 6 hours with the addition of acetate at the final concentration of 100 mg-C L^−1^. The aerobic condition was maintained by supplying air using an air pump at a flow rate of 0.5 L min^−1^ and the biomass suspension was continuously mixed with a magnetic stirring bar at 150 rpm. The pH was controlled to pH 8.0–8.2 during incubation by adding 1 N H_2_SO_4_ or 1 N NaOH. A liquid sample was collected every hour and the acetate concentration was determined. Supplementary acetate was added when the acetate concentration dropped below 40 mg-C L^−1^. The biomass suspension after 6 h of incubation was subjected to microbial analysis and the determination of PHA concentration.

### Dual staining with NBA and DAPI

Dual staining with NBA and DAPI was performed as previously described (47). Briefly, the biomass was sonicated at 3 watt for 4 min on ice with a Branson Sonifier 250D (Branson Ultrasonics, Danbury, CT, USA), and placed on glass slides (HTC super cured CEL-LINE series; Erie Scientific, Portsmouth, UK). The micro-organisms on the glass slides were first stained with NBA (0.1% w/v ethanol solution, certified dye content 81%, Kodak, NY, USA) for 30 min (31), and then with 2 μg mL^−1^ DAPI for 5 min.

### FISH analysis and post-FISH NBA staining

Fixation of biomass and *in situ* hybridization of oligonucleotide probes were performed as previously described (3, 30). The biomass was fixed in either 4% paraformaldehyde for Gram-negative organisms or 50% ethanol for Gram-positive organisms, sonicated at 3 watt for 4 min, and placed on the glass slides. After hybridization with the oligonucleotide probes shown in Table 1, the specimen was subjected to microscopy. After microscopy, post-FISH NBA staining was performed as previously described (56). The cover slip was carefully removed, the microorganisms on the slide were stained with NBA, and the same microscopic field was relocated and examined. The oligonucleotide probes used in the present study were synthesized and labeled at the 5′ end with the indocarbocyanine dye (Cy3) from the SIGMA Genosys (Ishikawa, Japan).

### PCR-cloning-sequencing analysis

Total genomic DNA was extracted from AS-A, AS-B, AS-E and AS-G by a FastDNA SPIN DNA extraction kit (MP Biomedicals LLC, OH, USA) following the instruction manual. The nearly full-length 16S rRNA gene sequence was amplified with 27f and 1492r (34) primer sets in 50-μL reaction tubes. Each reaction tube contained 1 × AmpliTaq Gold PCR reaction buffer, 0.2 mM deoxyribonucleotide triphosphate, 1.25 U AmpliTaq Gold (Life Technologies, CA, USA), 0.2 μM primer, and 50 ng extracted DNA. Thermal conditions of PCR amplification were as follows: initial denaturation at 95°C for 10 min, 20 cycles of denaturation at 94°C for 30 s, annealing at 50°C for 30 s and elongation at 72°C for 2 min, and final elongation at 72°C for 10 min. Fragment size of the amplicon was checked by 1.0% agarose gel electrophoresis. The amplicon was purified using a QIAquick PCR purification kit (Qiagen, Hilden, Germany), ligated into a pMD20-T vector by a Mighty cloning kit (Takara Bio, Otsu, Japan), and transformed into the cells of *Escherichia coli* K12 DH5α. Clones were randomly picked up and subjected to sequencing analysis using 357f primer (34) and an Applied Biosystems 3730 DNA analyzer (Life Technologies). The nucleotide bases assigned a Phred quality score lower than 15 were trimmed by the software Paracel Filtering Package (Paracel, CA, USA), and the sequence reads more than 300 bp were subjected to phylogenetic analysis. The phylogenetic affiliation was examined using the blastn search program (2) with the database of all non-redundant nucleotide sequences in the National Center for Biotechnology Information. Clones with greater than 95% sequence similarity were grouped into operational taxonomic units (OTUs), and the diversity indices including Shannon, Simpson and Chao1 and sampling coverage indices were calculated for each clone library by the software Mothur (55). For OTU-1–OTU-27, which were affiliated to class *α*- or *Betaproteobacteria*, nearly full-length 16S rRNA gene sequences were determined from representative clones of each OTU. If OTU contained clones derived from different activated sludge samples, representative clones from each activated sludge sample were selected and subjected to sequencing analysis. Sequencing analysis was conducted using M13 sequencing primers (forward and reverse) and 357f, 968f, 1099f, 518r, 907r and 1114r primers (34), the sequence reads were assembled by the software AutoAssembler (Life Technologies), and the vector sequences and annealing sites of 27f and 1492r primers were trimmed manually. The phylogenetic tree was developed using ARB software (37). First, the nucleotide sequences were imported into the SILVA database (SSURef_ 102_SILVA_12_02_10), aligned by the Integrated Aligners tool with the default parameters, and alignments were refined manually. The phylogenetic tree was constructed by the maximum parsimony (Phylip DNAPARS), neighbor-joining (Jukes-Cantor model) and maximum likelihood (RAxMX) methods. Bootstrap resampling analysis was performed using 1,000 replicates for the neighbor-joining method and 100 replicates for the maximum parsimony and maximum likelihood methods to estimate the confidence of tree topologies.

### Oligonucleotide probe design and optimization of formamide concentration

Oligonucleotide probes were designed using the Design probes tool in ARB software with the default parameters, and the coverage and specificity were first examined by comparative analysis of all sequences in the ARB database, which is composed of both the SILVA database and our clone sequences. The coverage and specificity of the oligonucleotide probes were further examined by the Probe Match tool in the Ribosomal Database Project (12).

The optimal formamide (FA) concentrations of oligonucleotide probes were determined by hybridization with a set of reference pure cultures or activated sludge samples, shown in Table S2. In this experiment, FA concentrations were changed in 5% increments, starting at 0% FA. The FA concentration, at which the fluorescence of the oligonucleotide probe was confirmed by microscopy from the biomass of a positive control but not of a negative control, was chosen as the optimal FA concentration.

### Microscopy

An Olympus BX51, equipped with a CCD camera DP70 (Olympus, Tokyo, Japan), was used for microscopy. DAPI fluorescence signals were observed through a WU filter, and NBA fluorescence signals were observed through a WIG filter (Olympus). The Cy3 and NBA fluorescence signals before or after post-FISH NBA staining were observed through WIG and NIBA filters, respectively. At least ten randomly selected fields containing more than one thousand total cells were used for the enumeration of PHAAOs. The numbers of total cells and the cells of PHAAOs were counted manually on the captured images.

### Chemical analysis

The concentration and monomer compositions of PHA in activated sludge were determined by gas chromatography (59). Briefly, the biomass was lyophilized, PHA was extracted and derivatized by acidic methanol, and the monomeric units of PHA were determined by gas chromatography. Sodium 3-hydroxybutyrate (Tokyo Chemical Industry, Tokyo, Japan) and a copolymer composed of 81% 3-hydroxybutyrate and 19% 3-hydroxyvalerate (Sigma-Aldrich) were used as the standards for 3-hydroxybutyrate and 3-hydroxyvalerate units, respectively.

The concentration of acetate was monitored by ion chromatography (ion chromatograph DX-A1100 equipped with an AS-9HC column; Thermo Fisher Scientific, MA, USA). The liquid sample was filtered through a 0.2 μm pore-size cellulose acetate membrane (Millipore, MA, USA) and the filtrates were injected to ion chromatography.

The concentration of MLVSS was determined according to the standard methods of the American Public Health Association (4).

### Linear regression analysis

Linear regression analysis was performed to identify the parameters that possibly caused the difference in the PHA accumulation rate among the activated sludge samples. The correlations were examined using Microsoft Excel 14.0.0.

### Nucleotide sequence accession numbers

Sequence data of 16S rRNA gene were deposited in the DDBJ nucleotide sequence database under accession numbers AB515437– AB516239.

## Results

### PHA accumulation by activated sludge

The activated sludge samples were aerobically incubated with the addition of acetate. PHA accumulated concurrently with the consumption of acetate, whereas no PHA accumulation was observed when the activated sludge samples were incubated without the addition of acetate. As shown in Table 2, the PHA accumulation rate and the conversion ratio of acetate into PHA were in the range of 8.5–38.2 mg-C g-VSS^−1^ h^−1^ and 29–64%, respectively. The increase of biomass concentration (excluding the amounts of PHA) during the batch experiment was less than 5%, indicating that the growth of microorganisms would be negligible.

### Abundance of PHAAOs in activated sludge

**Fig. 1:**
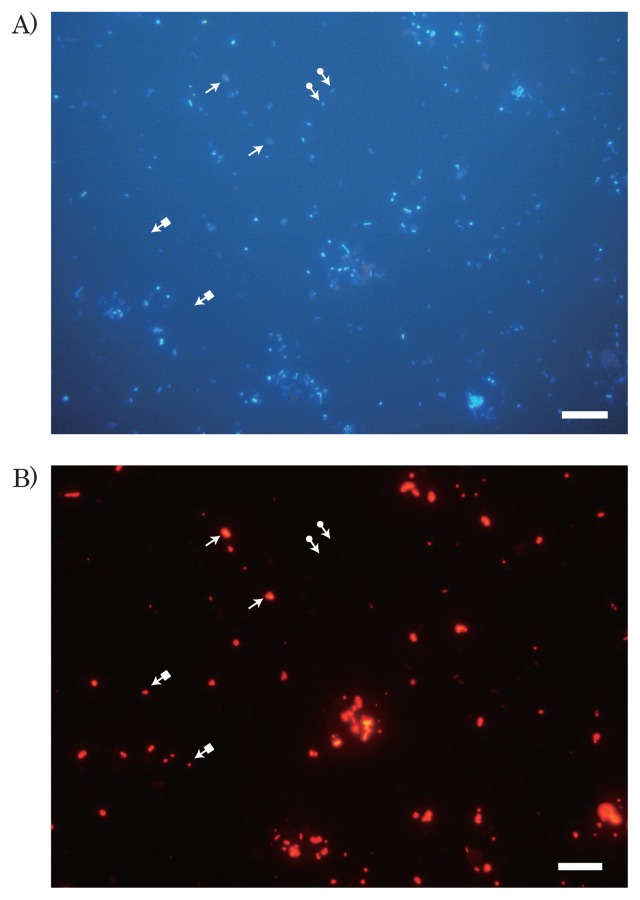
Micrographs taken after dual staining with DAPI and NBA. A) DAPI image, B) NBA image. The activated sludge sample accumulating PHA was stained with DAPI and NBA, and then examined by microscopy. Images A) and B) were taken at the same location. The following three particle types were found in these images and their examples are indicated by arrows: i) particles detected with only DAPI (arrows with a closed circle symbol at the end); ii) particles detected with DAPI and NBA (arrows without any symbol at the end); and iii) particles detected with only NBA (arrows with a closed square at the end). Bar=10 μm.

The abundance of PHAAOs in biomass was examined by microscopy after dual staining with NBA and DAPI. A typical microscopic image taken after dual staining with NBA and DAPI is shown in Fig. 1. The following three types of particles were observed in the biomass: i) particles exhibiting only the DAPI signal; ii) particles exhibiting both DAPI and NBA signals; and iii) particles exhibiting only the NBA signal. These particles were referred to as non-PHAAOs, PHAAOs, and NBA particles, respectively. In the present study, the total number of cells was defined as the sum of cells exhibiting the DAPI signal, which included the population of non-PHAAOs and PHAAOs. On the other hand, the abundance of NBA particles was ignored in the enumeration of PHAAOs and total cells.

We identified the population of PHAAOs from all activated sludge samples. The abundance of PHAAOs in the total number of cells ranged between 11% (AS-D) and 18% (AS-F), as presented in Table 3.

### Microbial community compositions of PHAAOs

The microbial community composition of PHAAOs was investigated by FISH analysis and post-FISH NBA staining techniques. A set of bacterial domain/phylum/class/order-specific oligonucleotide probes was first applied to outline the microbial community composition of PHAAOs. As shown in Table 4, the PHAAOs hybridized with the EUB mix probe accounted for 80% to more than 95% in the entire population of PHAAOs. The abundance of *α*- and *β*-proteobacterial PHAAOs was 13–40% and 53–83% in the entire population of PHAAOs, respectively. Members of PHAAOs affiliated to the class *Gammaproteobacteria*, the phylum *Bacteroidetes*, *Actinobacteria* or *Firmicutes* were also detected, while their abundance was less than 6% in the entire population of PHAAOs.

**Fig. 2:**
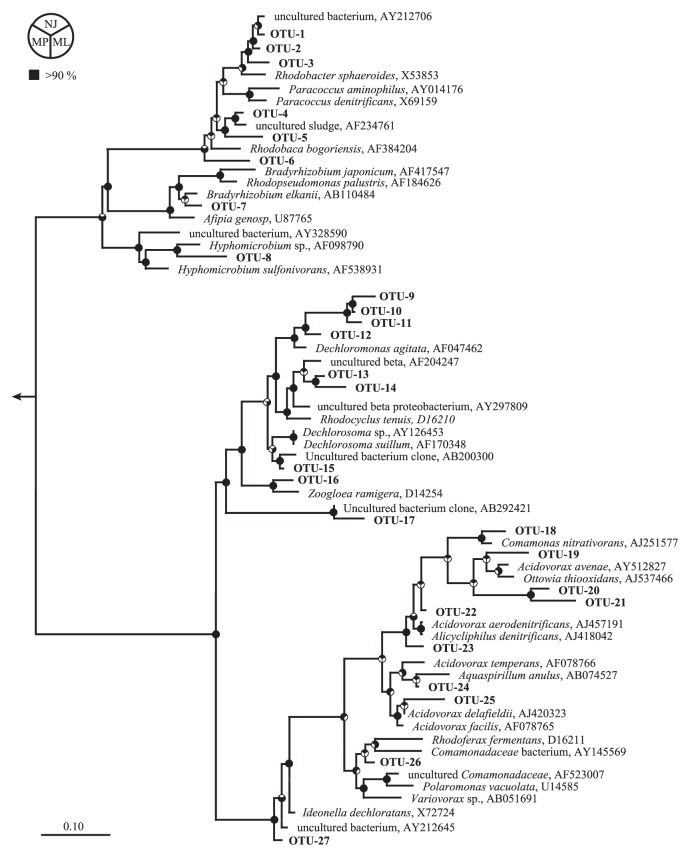
Maximum-parsimony tree based on the 16S rRNA gene sequences. The tree indicates the phylogenetic affiliation of OTUs in the class *Alphaproteobacteria* and *Betaproteobacteria* and their closest proteobacteria relatives. Nearly full-length 16S rRNA gene sequences of the representative clones in each OTUs were determined and aligned with their relatives in the SILVA database by the tool Integrated Aligners in ARB software. The phylogenetic tree was calculated by the maximum parsimony (Phylip DNAPARS), neighbor-joining (Jukes-Cantor model) or maximum likelihood (RAxMX) methods, and the phylogenetic tree calculated with the maximum parsimony method is shown here. Pie charts at the nodes represent the confidence of branch topology, and bootstrap values greater than 90% are in black (neighbor-joining method, NJ, for the upper sector; maximum parsimony method, MP, for the bottom-left sector; maximum likelihood method, ML, for the bottom-right sector). Scale bar represents 0.10 substitutions per nucleotide position.

The above community structure analysis revealed that dominant PHAAOs are members of the class *Alphaproteobacteria* or *Betaproteobacteria*, leading us to further investigate the microbial community structure of *α*- or *β*-proteobacterial PHAAOs. For this purpose, 16S rRNA gene clone libraries were constructed from AS-A, AS-B, AS-E and AS-G that exhibited low (8.5 and 10.9 mg-C g-VSS^−1^ h^−1^ for AS-A and AS-B, respectively), medium (19.7 mg-C g-VSS^−1^ h^−1^ for AS-E) and high (38.2 mg-C g-VSS^−1^ h^−1^ for AS-H) PHA accumulation rates in the batch experiments (Table 2). In total, 335 clones were randomly picked up from the four clone libraries and the 16S rRNA gene sequences were partially determined. The number of clones, OTUs, Shannon, Simpson, Chao1 and coverage indices of the four clone libraries are summarized in Table S3. The coverage indices of the four clone libraries ranged between 63 and 78%. We identified 8 and 19 OTUs in the class *Alphaproteobacteria* or *Betaproteobacteria*, respectively, and nearly the full length of 16S rRNA gene sequences was determined from the representative clones in each OTU. Phylogenetic relationships of the OTUs in class *Alphaproteobacteria* or *Betaproteobacteria* are presented in Fig. 2.

To identify PHA accumulation by microorganisms affiliated into the OTUs in class *Alphaproteobacteria* or *Betaproteobacteria*, 12 oligonucleotide probes were designed as follows: ARR994 for OTU-1, 2, and 3, ARP653 for OTU-4, 5, and 6, ABJ1302 for OTU-7, an AHS576 for OTU-8, BRDA454 for OTU-15, BCC1212 for OTU-18, BCO395 for OTU-19, 20 and 21, BCAD1422 for OTU-22 and 23, BCAT1010 for OTU-24 and 25, BCR622 for OTU-26 and BCI823 for OTU-27. The concentration of FA for each designed oligonucleotide probe is shown in Table 1 and was determined using the reference pure cultures or the activated sludge samples (Table S2). In addition, the oligonucleotide probes DEN441 for OTU-9, 10, 11 and 12, PAO846 for OTU-13 and 14, ZOO834 for OTU-16 and OTU1 mix for OTU-17 were also employed from previous reports (13, 19, 47). Even though ARR994, ARP653, DEN441, PAO846, BCO395, BCAD1422 and BCAT1010 probes are not specific to a single OTU but cover multiple OTUs, these oligonuleotide probes are useful for comparison of the microbial community composition of PHAAOs among the activated sludge samples.

The microbial community composition of PHAAOs was investigated by FISH analysis with a set of newly designed and selected oligonucleotide probes and post-FISH NBA staining techniques. As shown in Table 4, PHA accumulation was confirmed for the following OTUs: OTU-1, 2, 3, 4, 5 and 6 in the class *Alphaproteobacteria* and OTU-13, 14, 15, 16, 17, 19, 20, 21, 24, 25 and 26 in the class *Betaproteobacteria*. Their abundance ranged from <1% to 16%, which was quite different across the activated sludge samples. For instance, the PHAAOs in OTU-26 accounted for 16% in the entire population of PHAAOs in AS-A, whereas they were less than 1% in AS-B and AS-E, and even not detected in AS-G. This outcome clearly demonstrated that the microbial community compositions of *α*- and *β*-proteobacterial PHAAOs were different among the WWTPs.

## Discussion

The removal of biodegradable organic matter is the prime objective of biological wastewater treatment. Previous studies revealed that up to 45% of easily biodegradable organic matter in sewage was removed and tentatively stored in microbial cells as PHA granules (11, 48). In the present study, we incubated activated sludge samples with the addition of acetate and determined the PHA conversion ratio. As shown in Table 2, the PHA conversion rates obtained in the present study were in the range of 29–64%, and an even higher PHA conversion rate was determined from the activated sludge sample collected from a laboratory scale sequencing batch reactor (*i.e.* 66–almost 100%) (6). These outcomes indicate that large amounts of easily biodegradable organic matter can be removed by PHAAOs in activated sludge. On the other hand, PHAAOs were not as abundant in the activated sludge samples (11–18% in all cells) as in the PHA conversion rates. This finding suggests that the specific acetate uptake rate of PHAAOs was higher than that of non-PHAAOs. We estimated the cell-specific acetate uptake rates of PHAAOs and non-PHAAOs in activated sludge and pointed out that PHAAOs can exhibit a 1.5-fold higher acetate uptake rate (5.3–8.0 × 10^−10^ and 2.8–4.2 × 10^−10^ mgC cell^−1^ h^−1^ for PHAAOs and non-PHAAOs, respectively) (49). These observations support the idea that PHAAOs in activated sludge significantly contributed to the removal of acetate, a major fraction of volatile fatty acids in sewage (24, 51).

Despite the contribution of PHAAOs to the removal of biodegradable organic matter, their abundance and microbial community composition in activated sludge are still poorly understood, especially in full-scale WWTPs. So far, only one report is available, which described the microbial community composition of PHAAOs in full-scale WWTPs operated in AO and A2O mode (47). However, the 16S rRNA gene-based clone library (58, 62), PCR-denaturing gradient gel electrophoresis (DGGE) (9), and quinone profiling (23, 26) analysis have revealed that the microbial community composition in full-scale WWTPs operated in AO or A2O mode differed from that in fully aerobic mode; therefore, we investigated the microbial community compositions of PHAAOs in full-scale WWTPs operated in fully aerobic mode, the most common configuration mode of activated sludge processes throughout the world.

The outcome of FISH analysis and post-FISH NBA staining revealed that the population of PHAAOs in full-scale WWTPs was dominantly composed of the members of *α*-and *β*-proteobacterial PHAAOs. The dominance of *β*-proteobacterial PHAAOs was also reported in a lab-scale aerobic sequencing batch reactor (SBR) fed with acetate (15, 35, 57). Dionisi *et al.* (15) examined microbial community composition in activated sludge exhibiting high PHA-accumulating capacity (*i.e.* 392 mgCOD gCOD^−1^ h^−1^) by 16S rRNA gene-based clone library. Their outcome pointed out that the clones affiliated to the genera *Thauera, Alcaligenes* and *Comamonas* (*Betaproteobacteria*) were dominant and accounted for 76% in the clone library. In addition, the combination of FISH analysis and post-FISH NBA staining revealed the dominance of *β*-proteobacterial PHAAOs affiliated to the genus *Azoarcus* (23.3–45.9% in the entire pouplation of PHAAOs) or *Thauera* (41.1–49.4%) (35, 57). Moreover, we recently reported the dominance of *β*-proteobacterial PHAAOs in full-scale WWTPs operated in AO or A2O mode (39–60% in the entire population of PHAAOs) (47). The dominance of *α*-proteobacterial PHAAOs affiliated to the genus *Amaricoccus* (61% in the entire population of PHAAOs) has been also identified in activated sludge collected from a lab-scale SBR fed with propionate (35). On the other hand, the occurrence of *γ*-proteobacterial PHAAOs has been reported previously from an aerobic SBR with relatively short biomass retention time (*i.e.* 1 day) (27). In addition, the capability for the synthesis of intracellular PHA granules has been confirmed using microorganisms affiliated to the phylum *Firmicutes* (29), *Actinobacteria* (40) and *Chloroflexi* (33); however, PHAAOs affiliated to class *Gammaproteobacteria*, phylum *Firmicutes*, *Actinobacteria* and *Chloroflexi* were minor in full-scale WWTPs operated in fully aerobic mode, as shown in Table 4 (<6% in the entire population of PHAAOs).

Unexpectedly, NBA particles were found in activated sludge as the outcome of NBA staining, and were stained with NBA but not DAPI. Recently, the authors examined the characteristics of NBA particles, and concluded that they were not PHA-accumulating cells but PHA granules for the following reasons: (i) no fluorescent signal was obtained from NBA particles even after staining with nucleic acid-staining dyes, including DAPI, SYBR Green I or SYBR Gold (Invitrogen, OR, USA), (ii) no cellular structure was observed from NBA particles under electron microscopy and (iii) the buoyant density of NBA particles (1.21–1.23 gml^−1^) was significantly higher than that of microbial cells in activated sludge (1.16–1.18 gml^−1^) but comparable with that of pure PHA granules (1.21–1.23 gml^−1^) (Oshiki, *et al.*, unpublished data). These observations support the idea that the NBA particles were not PHA-accumulating cells but probably PHA granules, and thus the abundance of NBA particles was ignored in the enumeration of PHAAOs or total cells in the present study.

In contrast to the similarity of the microbial community composition of PHAAOs at domain/phylum/class/order level, the microbial community compositions of *α*- and *β*-proteobacterial PHAAOs differed among AS-A, AS-B, AS-E and AS-G. Such a difference was expected because a broad range of PHA accumulation rate (8.5–25.3 mg-C g-VSS^−1^ h^−1^) was found in the batch experiment even though the abundance of PHAAOs in these activated sludge samples was not significantly different (13–16% in the number of total cells). Therefore, it was speculated that the microbial community structure of *α*- and *β*-proteobacterial PHAAOs was different, which resulted in different PHA accumulation rates among the activated sludge samples. Various previous studies support this hypothesis as they have reported a wide range of PHA accumulation capability from pure isolates of PHAAOs (20, 61). For instance, PHA accumulation rates of *Cupriavidus necator*, *Azohydromonas lata*, *Azohydromonas australica*, *Burkholderia cepacia* and *Pelomonas saccharophila* have been reported to be in the range of 0.001–0.105 g-PHA g-cell-dry weight^−1^ h^−1^ (20).

Environmental conditions (*i.e.* wastewater composition and operational conditions) must affect the niche speciation of PHAAOs in full-scale WWTPs. As shown in Table S1, the wastewater composition and operational conditions differed among the WWTPs. For instance, the values of SRT ranged between 3 and 8 days among the 8 WWTPs. The influence of SRT on the microbial community structure in activated sludge has been investigated previously by 16S rDNA terminal restriction fragment length polymorphism (T-RFLP) (54) or DGGE (1) analysis, indicating that the bacterial diversity in activated sludge was influenced by the SRT values in the range of 2–8 days (54) or 2.6–10.4 days (1), which is comparable to the present study (3–8 days). The specific influence of SRT on the microbial community composition of PHAAOs is still unclear, while it could be a cause for the occurrence of different microbial community composition of PHAAOs in WWTPs. In addition, physiological characteristics of PHAAOs are another factor for deciding their niche speciation. Different physiological characteristics (*i.e.* optimal temperature for growth, maximum growth rate, substrate specificity and PHA production capability) have been demonstrated from the 6 pure cultures of PHAAOs, although the similarities of 16S rDNA nucleotide sequences implied that they were affiliated with the same species (25); however, further investigation is required to elucidate the specific influence of environmental conditions or physiological characteristics on the niche speciation of PHAAOs.

Interestingly, a weak but significant correlation was found between the values of SRT and PHA accumulation rates (Fig. S1, *R*^2^=0.61, *p*=0.05). The activated sludge sample collected from the WWTP operated with shorter SRT showed a higher PHA accumulation rate. In addition, the authors found a significant correlation between the population of *α*-proteobacterial PHAAOs in total PHAAOs and the PHA accumulation rates (Fig. S1, *R*^2^=0.66, *p*=0.05). These observations suggested that the operation at short SRT induced the proliferation of *α*-proteobacterial PHAAOs who had higher PHA accumulation capacity. The increase of PHA accumulation capacity of activated sludge under short SRT operational conditions was previously observed in the laboratory scale sequencing batch reactor, while the dominant PHAAOs were affiliated to *Gammaproteobacteria* and not *Alphaproteobacteria* (27). In the present study, the authors identified *α*-proteobacterial PHAAOs that were affiliated to OTU-1, 2, 3, 4, 5 or 6, while they were minor in the population of *α*-proteobacterial PHAAOs (Table 4). Previously, *α*-proteobacterial PHAAOs affiliated to the genus *Amaricoccus* have been identified from activated sludge (32) while their abundance was less in our activated sludge samples since tetrad-forming microorganisms, which are the typical cell morphology of the genus *Amaricoccus*, were not found under microscopy. Further investigation is essential to clarify the phylogeny of *α*-proteobacterial PHAAOs in full-scale WWTPs.

FISH analysis and post-FISH NBA staining allowed us to identify the members of PHAAOs affiliated to the following OTUs: OTU-1, 2, 3, 4, 5, 6, 13, 14, 15, 16, 17, 19, 20, 21, 24, 25 or 26. As for the members affiliated to OTU-13, 14, 15, 16 or 17, we previously identified their PHA accumulation in a similar manner in the present study from activated sludge samples collected from full-scale WWTPs operated in AO or A2O mode (47). This finding suggested that the members affiliated to OTU-13, 14, 15, 16 or 17 are distributed in full-scale WWTPs operated in fully-aerobic, AO and A2O mode. On the other hand, PHA accumulation by members affiliated to OTU-1, 2, 3, 4, 5, 6, 19, 20, 21, 24, 25 or 26 has not been demonstrated, while the PHA accumulation capacity by members affiliated to OTU-1, 2, 3 can be speculated from the knowledge of previous pure culture studies. The nucleotide sequences affiliated to OTU-1, 2 or 3 shared similarities with those of *Rhodobacter sphaeroides* (Accession number: X53853), which accumulate intercellular PHA granules (8). It should be noted that the distribution of PHAAOs in natural or man-made ecosystems is still poorly understood, although the accumulation of PHA has been demonstrated in various ecosystems such as soil (28, 53), compost (53), river biofilm (17), marsh microbial mats (53), algal mat (10), estuarine sediment (16, 21, 45) and freshwater (36, 60). In order to examine the distribution of PHAAOs identified in the present study, future studies are required.

Consequently, the present study demonstrated the distribution of PHAAOs in full-scale WWTPs operated in fully aerobic mode and also their significant contribution to the removal of acetate. The population of PHAAOs was mostly composed of *α*- and *β*-proteobacterial PHAAOs, whereas their microbial community compositions differed among the WWTPs. This study shed light on the microbial diversity of PHAAOs in full-scale WWTPs, and further studies are required to investigate the niche speciation of PHAAOs in activated sludge.

## Supplementary Material



## Figures and Tables

**Table 1:** Oligonucleotide probes used in the study. Phylogenetic affiliations of the OTUs defined in the study are shown in Fig. 2.

Probe name	Specificity	Sequence (5′-3′)	FA	Reference
EUB338^a^	Most Bacteria	GCTGCCTCCCGTAGGAGT	35%	(3)
EUB338-II^a^	*Planctomycetales*	GCAGCCACCCGTAGGTGT	35%	(14)
EUB338-III^a^	*Verrucomicrobiales*	GCTGCCACCCGTAGGTGT	35%	(14)
ALF968	*Alphaproteobacteria*	GGTAAGGTTCTGCGCGTT	20%	(43)
BET42a^e^	*Betaproteobacteria*	GCCTTCCCACTTCGTTT	35%	(38)
GAM42a^e^	*Gammaproteobacteria*	GCCTTCCCACATCGTTT	35%	(38)
HGC69a	*Actinobacteria*	TATAGTTACCACCGCCGT	35%	(52)
LGC354A^b^	*Firmicutes*	TGGAAGATTCCCTACTGC	35%	(41)
LGC354B^b^	*Firmicutes*	CGGAAGATTCCCTACTGC	35%	(41)
LGC354C^b^	*Firmicutes*	CCGAAGATTCCCTACTGC	35%	(41)
CF319a	*Bacteroidetes*	TGGTCCGTGTCTCAGTAC	35%	(39)
CFX1223	*Chloroflexi*	CCATTGTAGCGTGTGTGTMG	35%	(7)
ARR994^d^	OTU-1, 2, 3	GGTCCCGCGATACCCATG	40%	This study
Comp994		GGCCCCGCGATACCCATG		This study
ARP653^d^	OTU-4, 5, 6	CTCACCTCTCTCGAACTC	50%	This study
Comp653		CTCACCTCTCTCGACCTC		This study
ABJ1302	OTU-7	GTTGCAGAGCCCAATCCG	30%	This study
AHS576^d^	OTU-8	ACAAATCCGCCTACGTGC	55%	This study
Comp576		ATAAATCCGCCTACGTGC		This study
DEN441	OTU-9, 10, 11, 12	TGCGATTTCTTCCCGGCC	40%	(19)
PAO846	OTU-13, 14	GTTAGCTACGGCACTAAAAGG	35%	(13)
BRDA454	OTU-15	CCCCGTATTAGGAGATGCG	10%	This study
ZOO834	OTU-16	CTCAATGAGTCTCCTCACCG	50%	(47)
OTU1-427^c^	OTU-17	CCCGGACTAAAGCGGTTTAC	30%	(47)
OTU1-472^c^	OTU-17	TCCAGTACCATCAAAGCACG	30%	(47)
BCC1212	OTU-18	GTTTCTAGCCCCACCTAT	40%	This study
BCO395^e^	OTU-19, 20, 21	TTCATCCTGCACGCGGAATG	30%	This study
Comp395		TTCATCCTGCACGCGGCATG		This study
BCAD1422	OTU-22, 23	ACCCACTTCTGGCGAGAC	30%	This study
BCAT1010^d^	OTU-24, 25	CGAGCACTCCTCTATCTCTA	30%	This study
Comp1010		CGAGCACCCCTCTATCTCTA		This study
BCR622	OTU-26	GTCAGTACAGGTCCAGGGGA	40%	This study
BCI823	OTU-27	AACCCCTCCAACAACCAGTT	10%	This study

a Used in an equimolar, EUB mix,

b Used in an equimolar, LGC mix,

c Used in an equimolar, OTU1 mix,

d Used in combination with their corresponding unlabeled competitor probes

**Table 2:** PHA accumulation rates and conversion rates of acetate into PHA. Activated sludge samples were aerobically incubated with the addition of 100 mg-C l^−1^ acetate. After 6 h of incubation, the acetate and PHA concentrations were determined by ion chromatography and gas chromatography, respectively. PHA conversion ratio was calculated by dividing the amount of accumulated PHA by the amount of consumed acetate

Activated sludge sample	PHA accumulation rate (mg-C g-VSS^−1^ h^−1^)	PHA conversion ratio (mg-C mg-C^−1^ × 100)
AS-A	8.5	39%
AS-B	10.9	29%
AS-C	11.5	41%
AS-D	11.6	35%
AS-E	19.7	49%
AS-F	20.1	40%
AS-G	25.3	45%
AS-H	38.2	64%

**Table 3:** Abundance of PHAAOs in activated sludge samples. Activated sludge samples were stained with NBA and DAPI, and then examined by microscopy to enumerate the abundance of PHAAOs in the total number of cells. Errors indicate the range of s.d. derived from 10 randomly captured images

Activated sludge sample	PHAAOs/Total cells
AS-A	15 ± 5%
AS-B	13 ± 3%
AS-C	12 ± 3%
AS-D	11 ± 2%
AS-E	13 ± 2%
AS-F	18 ± 5%
AS-G	16 ± 2%
AS-H	14 ± 3%

**Table 4:** Microbial community composition of PHAAOs in the 8 full-scale WWTPs. Activated sludge samples were examined by FISH with the oligonucleotide probes described in Table 1 and post-FISH NBA staining analysis. Abundance of individual PHAAOs refers to the entire population of PHAAOs. n.d.: not detected

Target	Activated sludge sample							

	AS-A	AS-B	AS-C	AS-D	AS-E	AS-F	AS-G	AS-H
*Most bacteria*^a^	>95%	89 ± 14%	88 ± 11%	>95%	>95%	>95%	>95%	80 ± 8%
*Alphaproteobacteria*	14 ± 4%	13 ± 5%	17 ± 7%	27 ± 8%	15 ± 8%	18 ± 7%	30 ± 14%	40 ± 9%
OTU-1, 2, 3	<1%	<1%	—	—	n.d.	—	<1%	—
OTU-4, 5, 6	n.d.	<1%	—	—	2 ± 1%	—	n.d.	—
OTU-7	n.d.	n.d.	—	—	n.d.	—	n.d.	—
OTU-8	n.d.	n.d.	—	—	n.d.	—	n.d.	—
*Betaproteobacteria*	70 ± 6%	56 ± 12%	64 ± 14%	83 ± 13%	74 ± 14%	71 ± 15%	60 ± 16%	53 ± 10%
OTU-9, 10, 11, 12	n.d.	n.d.	—	—	n.d.	—	n.d.	—
OTU-13, 14	n.d.	<1%	—	—	9 ± 7%	—	n.d.	—
OTU-15	n.d.	2 ± 1%	—	—	15 ± 6%	—	3 ± 2%	—
OTU-16	5 ± 3%	4 ± 3%	—	—	7 ± 2%	—	3 ± 2%	—
OTU-17	8 ± 3%	4 ± 2%	—	—	2 ± 1%	—	<1%	—
OTU-18	n.d.	n.d.	—	—	n.d.	—	n.d.	—
OTU-19, 20, 21	5 ± 4%	7 ± 4%	—	—	13 ± 6%	—	4 ± 2%	—
OTU-22, 23	n.d.	n.d.	—	—	n.d.	—	n.d.	—
OTU-24, 25	n.d.	5 ± 3%	—	—	4 ± 2%	—	<1%	—
OTU-26	16 ± 5%	<1%	—	—	<1%	—	n.d.	—
OTU-27	n.d.	n.d.	—	—	n.d.	—	n.d.	—
*Gammaproteobacteria*	3 ± 2%	4 ± 2%	2 ±1%	4 ± 2%	3 ± 1%	<1%	6 ± 2%	5 ± 2%
*Bacteroidetes*	3 ± 1%	5 ± 3%	<1%	2 ± 1%	3 ± 1%	n.d.	5 ± 2%	<1%
*Actinobacteria*	n.d.	n.d.	<1%	2 ± 2%	2 ± 2%	n.d.	<1%	n.d.
*Firmicutes*	n.d.	n.d.	n.d.	2 ± 1%	n.d.	n.d.	n.d.	n.d.
*Chloroflexi*	n.d.	n.d.	n.d.	n.d.	n.d.	n.d.	n.d.	n.d.

a Hybridized with the EUB mix probe

## Abbreviations

AO: anaerobic-oxic
A2O: anaerobic-anoxic-oxic
DAPI: 4′,6-diamidino-2-phenylindole
DGGE: denaturing gradient gel electrophoresis
FISH: fluorescence *in situ* hybridization
FA: formamide
GAOs: glycogen-accumulating organisms
MLVSS: mixed liquor volatile suspended solids
NBA: Nile blue A
OTU: operational taxonomic unit
PAOs: polyphosphate-accumulating organisms
PHA: polyhydroxyalkanoates
PHAAOs: PHA-accumulating organisms
SBR: sequencing batch reactor
SRT: sludge retention time
T-RFLP: terminal restriction fragment length polymorphism
WWTP: wastewater treatment plant
