# Supplementary material for: Microbial Community Composition of Polyhydroxyalkanoate-Accumulating Organisms in Full-Scale Wastewater Treatment Plants Operated in Fully Aerobic Mode
Source: Microbes Environ. 2012 Dec 19;28(1):96–104. doi: 10.1264/jsme2.ME12141 (PMC4070681; doi:10.1264/jsme2.ME12141)
Supplement: Supplementary file 1 [file 28_96_s1.pdf]

Table S1. Operational conditions and water qualities on the 8 WWTPs sampled in the present study.

Temperature and pH were determined in the aeration tanks whereas the measurement of BOD_5, COD, TN, ammonia, nitrite, nitrate and TP were performed with the influents.

WWTP	Temperature (°C)	pH	BOD_5 [a)] (mg L^{-1})	COD [b)] (mg L^{-1})	TN (mg L^{-1})	Ammonia (mg L^{-1})	Nitrite (mg L^{-1})	Nitrate (mg L^{-1})	TP (mg L^{-1})	MLVSS [c)] (mg-VSS L^{-1})	SRT [d)] (day^{-1})	HRT [e)] (day^{-1})	Sludge return ratio
A	19.9	6.8	170	110	29	14	0.1	0.2	3.6	870	8	0.37	37%
B	21	7.1	110	82	29	17	0.1	0.1	3.5	988	7	0.45	29%
C	19.8	7.1	160	84	22	15	0.1	0.1	3.3	1478	8	0.37	37%
D	20.7	7	230	98	32	20	0.1	0.1	3.8	1311	7	0.23	52%
E	20.6	6.9	190	100	43	28	0.1	0.1	4.2	1278	3	0.25	39%
F	21.3	7.1	170	91	27	17	0.2	0.2	3.3	731	4	0.26	44%
G	21	7.1	120	67	32	22	0.1	0.2	2.9	1166	6	0.35	32%
H	26.3	7.2	160	76	31	21	0.1	0.1	3	697	3	0.33	73%

[a)] Biological oxygen demand, [b)] Manganese (III) chemical oxygen demand, [c)] Mixed liquor volatile suspended solids, [d)] Sludge retention time, and [e)] Hydraulic retention time

Table S2. Summary of the microorganisms and activated sludge samples used for the optimization of the formamide concentration of newly designed oligonucleotide probes.

Probe name	Positive control	Negative control
ARR994	*Paracoccus aminovorans* (JCM 7865)	*Rhodobacter capsulatus* (JCM 21090)<br>*Rhodobacter blasticus* (NBRC 16437)<br>*Rhodocista centenaria* (JCM 21060T)
ARP653	*Paracoccus pantotrophus* (JCM 20620)	*Alicyclobacillus pomorum* (JCM 21459T)<br>*Rhodobacter blasticus* (NBRC 16437)<br>*Phenylobacterium immobile* (DSM 1986)
ABJ1302	*Bradyrhizobium japonicum* (JCM 20679)	*Arthrobacter globiformis* (IAM 12438)
AHS576	*Filomicrobium fusiforme* (DSM 5304)	*Sphingomonas parapaucimobilis* (JCM 7510)<br>*Starkeya novella* (JCM 20403)<br>*Hyphomicrobium methylovorum* (JCM 6890)
BRDA454	Activated sludge (AS-A)	-
BCC1212	*Comamonas denitrificans* (DSM 17887)	*Rhodoferax fermentans* (NBRC 16659)
BCO395	*Ottowia thiooxydans* (JCM 11629)	*Comamonas badia* (JCM 21345)
BCAD1422	*Alicycliphilus denitrificans* (DSM 14773)	*Delftia acidovorans* (IAM12409)
BCAT1010	*Ottowia thiooxydans* (JCM 11629)	*Rhodoferax fermentans* (NBRC 16659)
BCR622	*Albidiferax ferrireducens* (DSM 15236)	*Rubrivivax gelatinosus* (JCM 21318)<br>*Rhodoferax fermentans* (NBRC 16659)
BCI823	Activated sludge (AS-G)	-

Table S3. Diversity and coverage indices in the 16S rRNA gene clone libraries constructed from AS-A, AS-B, AS-E and AS-G.

Activated sludge sample	Clone numbers	OTUs	Shannon	Simpson	Chao1	Coverage
AS-A	86	27	2.2	0.26	84	78%
AS-B	79	40	3.1	0.09	98	63%
AS-E	86	34	2.7	0.14	134	71%
AS-G	84	38	2.8	0.15	74	69%

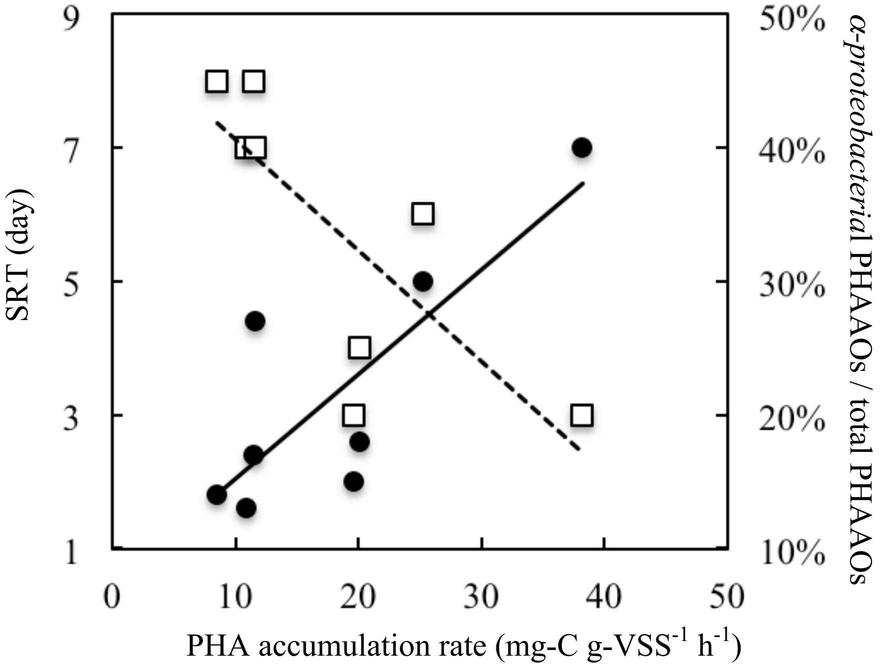

Fig. S1. Regression analysis between the PHA accumulation rates and SRTs or the abundances of *α-proteobacterial* PHAAOs in total PHAAOs: The authors investigated the correlations between the PHA accumulation rates and the operational parameters, the water qualities of full-scale WWTPs shown in **Table S1**, the abundances of PHAAOs in total cells (**Table 3**) or the abundances of each groups of PHAAOs (**Table 4**). Statistically significant correlations (student *t*-test, p=0.05) were only observed on the SRTs (open squares, $R^2 = 0.61$) and the abundances of *α-proteobacterial* PHAAOs in total PHAAOs (filled circles, $R^2 = 0.66$). The activated sludge samples collected from the full-scale WWTPs operated with shorter SRT showed the higher PHA accumulation rate. In addition, the higher abundance of *α-proteobacterial* PHAAOs caused the higher PHA accumulation rates of activated sludge samples.
